# (1*E*,4*E*)-1-(Thio­phen-2-yl)-5-(2,6,6-trimethyl­cyclo­hex-1-en-1-yl)penta-1,4-dien-3-one

**DOI:** 10.1107/S1600536812022465

**Published:** 2012-05-23

**Authors:** Ya-Li Zhang, Liu-Fang Xiang, Peng Zou, Yi-Jun Jin, Shu-Lin Yang

**Affiliations:** a Institute of Biotechnology, Nanjing University of Science and Technology, Nanjing, Jiangsu Province 210094, People’s Republic of China

## Abstract

In the title curcumin–ionone derivative, C_18_H_22_OS, the dihedral angle between the thia­zole ring and the mean plane through the cyclo­hexene ring is 5.16 (10)°. The mol­ecule has an *E* conformation for each of the olefinic bonds.

## Related literature

For related structures, see: Liang *et al.* (2007 ▶); Zou *et al.* (2012 ▶). For background to the biological properties of curcumin–ionone derivatives, see: Anand *et al.* (2008 ▶); Zhao *et al.* (2010*a* ▶,*b* ▶); Zhou *et al.* (2009*a* ▶,*b* ▶).
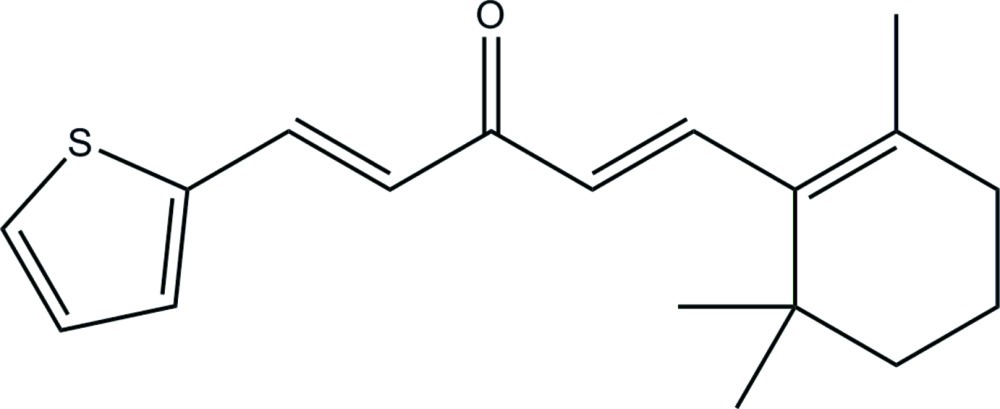


## Experimental

### 

#### Crystal data

C_18_H_22_OS
*M* *_r_* = 286.42
Monoclinic, 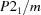

*a* = 8.3401 (17) Å
*b* = 6.9084 (14) Å
*c* = 13.994 (3) Å
β = 96.082 (4)°
*V* = 801.7 (3) Å^3^
*Z* = 2
Mo *K*α radiation
μ = 0.20 mm^−1^
*T* = 293 K
0.36 × 0.30 × 0.15 mm

#### Data collection

Bruker SMART CCD area-detector diffractometer
Absorption correction: multi-scan (*SADABS*; Bruker, 2002 ▶) *T*_min_ = 0.674, *T*_max_ = 1.000
4888 measured reflections
1711 independent reflections
1555 reflections with *I* > 2σ(*I*)
*R*_int_ = 0.020

#### Refinement

*R*[*F* ^2^ > 2σ(*F* ^2^)] = 0.046
*wR*(*F* ^2^) = 0.137
*S* = 1.05
1711 reflections
145 parameters
4 restraints
H atoms treated by a mixture of independent and constrained refinement
Δρ_max_ = 0.32 e Å^−3^
Δρ_min_ = −0.17 e Å^−3^

### 

Data collection: *SMART* (Bruker, 2002 ▶); cell refinement: *SAINT* (Bruker, 2002 ▶); data reduction: *SAINT*; program(s) used to solve structure: *SHELXS97* (Sheldrick, 2008 ▶); program(s) used to refine structure: *SHELXL97* (Sheldrick, 2008 ▶); molecular graphics: *SHELXTL* (Sheldrick, 2008 ▶); software used to prepare material for publication: *SHELXTL*.

## Supplementary Material

Crystal structure: contains datablock(s) I, global. DOI: 10.1107/S1600536812022465/ng5270sup1.cif

Structure factors: contains datablock(s) I. DOI: 10.1107/S1600536812022465/ng5270Isup2.hkl

Supplementary material file. DOI: 10.1107/S1600536812022465/ng5270Isup3.cml

Additional supplementary materials:  crystallographic information; 3D view; checkCIF report

## Figures and Tables

**Fig. 1.**
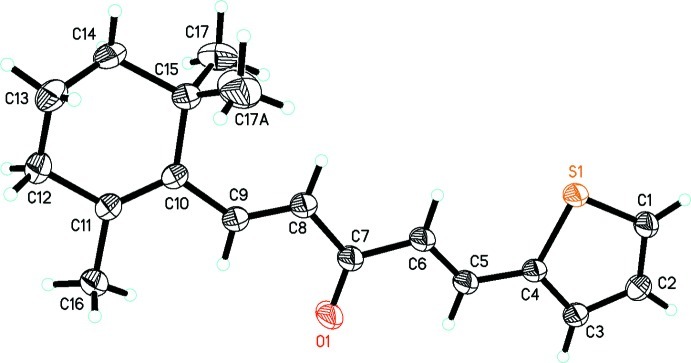
The molecular structure of the title compound, showing 30% displacement ellipsoids for the non-hydrogen atoms. Hydrogen atoms are drawn as spheres of arbitrary radius.

## supplementary crystallographic information

### Comment

The β-ionone unit is a phytochemical present in many fruit, vegetable and grain. It is found to exert *in vitro* anticarcinogenic and antitumor activities; ionone derivatives are valuable intermediates for the chemo-enzymatic synthesis of carotenoids, astaxanthin and zeaxanthin (Zhou *et al.*, 2009*a*,*b*). On the other hand, curcumin (diferuloylmethane) is a polyphenolic phytochemical found in turmeric (*Curcuma longa*) that has useful medicinal properties (Anand *et al.*, 2008). Previously, we have evaluated mono-carbonylanalogues of curcumin for anti-inflammatory properties and discussed structure-activity relationships (Liang *et al.*, 2007; Zhao *et al.*,2010*a*,*b*).

In the ionone-based curcumin title compound (Scheme I), all bond dimensions are normal. The dihedral angles between the thiazole ring and the cyclohexene ring is 5.16 (10)°.

### Experimental

To the mixture of β-ionone (2.5 mmol, 0.481 g) and thiophene-2-carbaldehyde (2.5mmol) in 10 ml EtOH, 1 ml of 10% NaOH was added and the mixture was stirred for 12 h at room temperature. After addition of 10 ml H_2_O, the solution was extracted by 3×10 ml CH_2_Cl_2_. The crude product obtained from the combined organic layers was purified by silica gel column chromatography (elutant: EtOAc/hexane). Crystals were obtained from an ethanol/EtOAc solution (1:2, v/v) at 293 K.

### Refinement

The H atoms were positioned geometrically (C—H = 0.93 and 0.96 Å) and refined as riding with *U*iso(H) = 1.2*U*eq(C) or 1.5*U*eq(methyl C).

### Crystal data

**Table:**

C_18_H_22_OS	*F*(000) = 308
*M**_r_* = 286.42	*D*_x_ = 1.186 Mg m^−^^3^
Monoclinic, *P*2_1_/*m*	Mo *K*α radiation, λ = 0.71073 Å
*a* = 8.3401 (17) Å	Cell parameters from 2746 reflections
*b* = 6.9084 (14) Å	θ = 4.9–56.5°
*c* = 13.994 (3) Å	µ = 0.20 mm^−^^1^
β = 96.082 (4)°	*T* = 293 K
*V* = 801.7 (3) Å^3^	Prismatic, colorless
*Z* = 2	0.36 × 0.30 × 0.15 mm

### Data collection

**Table:**

Bruker SMART CCD area-detector diffractometer	1711 independent reflections
Radiation source: fine-focus sealed tube	1555 reflections with *I* > 2σ(*I*)
Graphite monochromator	*R*_int_ = 0.020
phi and ω scans	θ_max_ = 26.0°, θ_min_ = 1.5°
Absorption correction: multi-scan (*SADABS*; Bruker, 2002)	*h* = −10→8
*T*_min_ = 0.674, *T*_max_ = 1.000	*k* = −8→8
4888 measured reflections	*l* = −17→16

### Refinement

**Table:**

Refinement on *F*^2^	Secondary atom site location: difference Fourier map
Least-squares matrix: full	Hydrogen site location: inferred from neighbouring sites
*R*[*F*^2^ > 2σ(*F*^2^)] = 0.046	H atoms treated by a mixture of independent and constrained refinement
*wR*(*F*^2^) = 0.137	*w* = 1/[σ^2^(*F*_o_^2^) + (0.091*P*)^2^ + 0.1463*P*] where *P* = (*F*_o_^2^ + 2*F*_c_^2^)/3
*S* = 1.05	(Δ/σ)_max_ = 0.002
1711 reflections	Δρ_max_ = 0.32 e Å^−^^3^
145 parameters	Δρ_min_ = −0.17 e Å^−^^3^
4 restraints	Extinction correction: *SHELXL97* (Sheldrick, 2008), Fc^*^=kFc[1+0.001xFc^2^λ^3^/sin(2θ)]^-1/4^
Primary atom site location: structure-invariant direct methods	Extinction coefficient: 0.013 (5)

### Special details

**Table:**

Geometry. All e.s.d.'s (except the e.s.d. in the dihedral angle between two l.s. planes) are estimated using the full covariance matrix. The cell e.s.d.'s are taken into account individually in the estimation of e.s.d.'s in distances, angles and torsion angles; correlations between e.s.d.'s in cell parameters are only used when they are defined by crystal symmetry. An approximate (isotropic) treatment of cell e.s.d.'s is used for estimating e.s.d.'s involving l.s. planes.
Refinement. Refinement of *F*^2^ against ALL reflections. The weighted *R*-factor *wR* and goodness of fit *S* are based on *F*^2^, conventional *R*-factors *R* are based on *F*, with *F* set to zero for negative *F*^2^. The threshold expression of *F*^2^ > σ(*F*^2^) is used only for calculating *R*-factors(gt) *etc*. and is not relevant to the choice of reflections for refinement. *R*-factors based on *F*^2^ are statistically about twice as large as those based on *F*, and *R*- factors based on ALL data will be even larger.

### Fractional atomic coordinates and isotropic or equivalent isotropic displacement parameters (Å^2^)

**Table:**

	*x*	*y*	*z*	*U*_iso_*/*U*_eq_	Occ. (<1)
S1	1.08493 (6)	0.2500	0.58526 (4)	0.0487 (3)	
O1	0.4327 (2)	0.2500	0.53246 (12)	0.0817 (7)	
C1	1.2154 (3)	0.2500	0.68697 (16)	0.0521 (6)	
H1	1.3270	0.2500	0.6874	0.063*	
C2	1.1384 (3)	0.2500	0.76737 (16)	0.0533 (6)	
H2	1.1919	0.2500	0.8292	0.064*	
C3	0.9688 (3)	0.2500	0.74818 (15)	0.0464 (5)	
H3	0.8982	0.2500	0.7954	0.056*	
C4	0.9204 (3)	0.2500	0.65023 (14)	0.0416 (5)	
C5	0.7575 (3)	0.2500	0.60586 (16)	0.0463 (5)	
H5	0.6772	0.2500	0.6472	0.056*	
C6	0.7078 (3)	0.2500	0.51249 (15)	0.0504 (6)	
H6	0.7839	0.2500	0.4685	0.060*	
C7	0.5361 (3)	0.2500	0.47694 (16)	0.0525 (6)	
C8	0.4939 (3)	0.2500	0.37222 (15)	0.0537 (6)	
H8	0.5754	0.2500	0.3317	0.064*	
C9	0.3412 (3)	0.2500	0.33499 (15)	0.0478 (5)	
H9	0.2683	0.2500	0.3809	0.057*	
C10	0.2646 (3)	0.2500	0.23645 (14)	0.0442 (5)	
C11	0.1024 (3)	0.2500	0.22224 (15)	0.0439 (5)	
C12	0.0090 (3)	0.2500	0.12463 (18)	0.0631 (7)	
C13	0.1137 (5)	0.3120 (7)	0.0454 (2)	0.0749 (18)	0.50
C14	0.2610 (5)	0.1876 (8)	0.0579 (2)	0.0745 (19)	0.50
C15	0.3698 (3)	0.2500	0.15222 (17)	0.0624 (7)	
C16	−0.0078 (3)	0.2500	0.30125 (17)	0.0516 (6)	
H16A	−0.0320	0.3810	0.3175	0.077*	0.50
H16B	0.0445	0.1857	0.3569	0.077*	0.50
H16C	−0.1060	0.1833	0.2796	0.077*	0.50
C17	0.4761 (3)	0.0695 (4)	0.15515 (17)	0.0931 (8)	
H17A	0.5515	0.0723	0.2119	0.140*	
H17B	0.5338	0.0669	0.0993	0.140*	
H17C	0.4099	−0.0440	0.1560	0.140*	
H14A	0.317 (4)	0.2500	0.005 (2)	0.091 (11)*	
H12A	−0.061 (3)	0.137 (3)	0.1184 (16)	0.077 (6)*	
H14B	0.253 (8)	0.046 (3)	0.061 (5)	0.13 (3)*	0.50
H13A	0.060 (4)	0.2500	−0.0122 (18)	0.096 (11)*	
H13B	0.159 (5)	0.445 (3)	0.048 (3)	0.052 (11)*	0.50

### Atomic displacement parameters (Å^2^)

**Table:**

	*U*^11^	*U*^22^	*U*^33^	*U*^12^	*U*^13^	*U*^23^
S1	0.0413 (4)	0.0712 (4)	0.0340 (3)	0.000	0.0062 (2)	0.000
O1	0.0398 (9)	0.168 (2)	0.0387 (9)	0.000	0.0092 (7)	0.000
C1	0.0394 (11)	0.0740 (16)	0.0423 (11)	0.000	0.0013 (9)	0.000
C2	0.0513 (13)	0.0721 (15)	0.0351 (11)	0.000	−0.0017 (9)	0.000
C3	0.0454 (12)	0.0565 (13)	0.0375 (10)	0.000	0.0053 (9)	0.000
C4	0.0409 (11)	0.0488 (11)	0.0356 (10)	0.000	0.0065 (8)	0.000
C5	0.0399 (11)	0.0601 (13)	0.0398 (11)	0.000	0.0088 (8)	0.000
C6	0.0403 (12)	0.0745 (16)	0.0372 (11)	0.000	0.0085 (9)	0.000
C7	0.0407 (12)	0.0787 (16)	0.0388 (11)	0.000	0.0069 (9)	0.000
C8	0.0451 (12)	0.0802 (17)	0.0364 (11)	0.000	0.0075 (9)	0.000
C9	0.0437 (11)	0.0646 (14)	0.0357 (10)	0.000	0.0066 (8)	0.000
C10	0.0453 (11)	0.0540 (12)	0.0333 (10)	0.000	0.0043 (8)	0.000
C11	0.0469 (11)	0.0470 (11)	0.0374 (10)	0.000	0.0026 (9)	0.000
C12	0.0506 (14)	0.093 (2)	0.0437 (13)	0.000	−0.0045 (11)	0.000
C13	0.071 (2)	0.115 (6)	0.0359 (15)	0.016 (2)	−0.0032 (14)	0.0065 (18)
C14	0.069 (2)	0.121 (6)	0.0340 (15)	0.018 (2)	0.0062 (14)	−0.0074 (17)
C15	0.0488 (13)	0.103 (2)	0.0362 (11)	0.000	0.0087 (10)	0.000
C16	0.0444 (12)	0.0626 (14)	0.0486 (12)	0.000	0.0089 (9)	0.000
C17	0.0879 (16)	0.113 (2)	0.0838 (15)	0.0131 (14)	0.0331 (12)	−0.0305 (14)

### Geometric parameters (Å, º)

**Table:**

S1—C1	1.698 (2)	C12—C13	1.543 (5)
S1—C4	1.724 (2)	C12—C13^i^	1.543 (5)
O1—C7	1.220 (3)	C12—H12A	0.97 (2)
C1—C2	1.353 (3)	C13—C13^i^	0.856 (10)
C1—H1	0.9300	C13—C14^i^	1.222 (6)
C2—C3	1.411 (3)	C13—C14	1.494 (6)
C2—H2	0.9300	C13—H13A	0.979 (18)
C3—C4	1.387 (3)	C13—H13B	0.994 (19)
C3—H3	0.9300	C14—C14^i^	0.862 (11)
C4—C5	1.433 (3)	C14—C13^i^	1.222 (6)
C5—C6	1.328 (3)	C14—C15	1.580 (4)
C5—H5	0.9300	C14—H14A	1.015 (18)
C6—C7	1.465 (3)	C14—H14B	0.99 (2)
C6—H6	0.9300	C15—C17	1.528 (3)
C7—C8	1.470 (3)	C15—C17^i^	1.528 (3)
C8—C9	1.324 (3)	C15—C14^i^	1.580 (4)
C8—H8	0.9300	C16—H16A	0.9600
C9—C10	1.457 (3)	C16—H16B	0.9600
C9—H9	0.9300	C16—H16C	0.9600
C10—C11	1.346 (3)	C17—H17A	0.9600
C10—C15	1.542 (3)	C17—H17B	0.9600
C11—C12	1.499 (3)	C17—H17C	0.9600
C11—C16	1.511 (3)		
			
C1—S1—C4	91.89 (10)	C13^i^—C13—H13A	64.1 (7)
C2—C1—S1	112.24 (18)	C14^i^—C13—H13A	119 (2)
C2—C1—H1	123.9	C14—C13—H13A	98.3 (16)
S1—C1—H1	123.9	C12—C13—H13A	103 (2)
C1—C2—C3	113.3 (2)	C13^i^—C13—H13B	158 (3)
C1—C2—H2	123.3	C14^i^—C13—H13B	68 (3)
C3—C2—H2	123.3	C14—C13—H13B	103 (3)
C4—C3—C2	111.63 (19)	C12—C13—H13B	118 (2)
C4—C3—H3	124.2	H13A—C13—H13B	125 (3)
C2—C3—H3	124.2	C14^i^—C14—C13^i^	89.9 (3)
C3—C4—C5	126.24 (19)	C14^i^—C14—C13	54.9 (3)
C3—C4—S1	110.90 (16)	C13^i^—C14—C13	35.0 (4)
C5—C4—S1	122.86 (16)	C14^i^—C14—C15	74.18 (19)
C6—C5—C4	127.5 (2)	C13^i^—C14—C15	126.7 (3)
C6—C5—H5	116.2	C13—C14—C15	109.5 (3)
C4—C5—H5	116.2	C14^i^—C14—H14A	64.9 (6)
C5—C6—C7	121.74 (19)	C13^i^—C14—H14A	115 (2)
C5—C6—H6	119.1	C13—C14—H14A	96.0 (16)
C7—C6—H6	119.1	C15—C14—H14A	103 (2)
O1—C7—C6	121.0 (2)	C14^i^—C14—H14B	175 (4)
O1—C7—C8	121.6 (2)	C13^i^—C14—H14B	86 (4)
C6—C7—C8	117.42 (19)	C13—C14—H14B	121 (4)
C9—C8—C7	120.7 (2)	C15—C14—H14B	106 (4)
C9—C8—H8	119.6	H14A—C14—H14B	119 (4)
C7—C8—H8	119.6	C17—C15—C17^i^	109.3 (3)
C8—C9—C10	132.8 (2)	C17—C15—C10	110.80 (14)
C8—C9—H9	113.6	C17^i^—C15—C10	110.80 (14)
C10—C9—H9	113.6	C17—C15—C14^i^	121.7 (2)
C11—C10—C9	118.22 (19)	C17^i^—C15—C14^i^	94.6 (2)
C11—C10—C15	122.08 (19)	C10—C15—C14^i^	108.4 (2)
C9—C10—C15	119.7 (2)	C17—C15—C14	94.6 (2)
C10—C11—C12	123.5 (2)	C17^i^—C15—C14	121.7 (2)
C10—C11—C16	124.87 (19)	C10—C15—C14	108.4 (2)
C12—C11—C16	111.65 (19)	C14^i^—C15—C14	31.6 (4)
C11—C12—C13	112.1 (2)	C11—C16—H16A	109.5
C11—C12—C13^i^	112.1 (2)	C11—C16—H16B	109.5
C13—C12—C13^i^	32.2 (4)	H16A—C16—H16B	109.5
C11—C12—H12A	109.2 (14)	C11—C16—H16C	109.5
C13—C12—H12A	122.5 (13)	H16A—C16—H16C	109.5
C13^i^—C12—H12A	95.3 (13)	H16B—C16—H16C	109.5
C13^i^—C13—C14^i^	90.1 (3)	C15—C17—H17A	109.5
C13^i^—C13—C14	54.9 (3)	C15—C17—H17B	109.5
C14^i^—C13—C14	35.2 (5)	H17A—C17—H17B	109.5
C13^i^—C13—C12	73.90 (18)	C15—C17—H17C	109.5
C14^i^—C13—C12	122.3 (3)	H17A—C17—H17C	109.5
C14—C13—C12	106.1 (3)	H17B—C17—H17C	109.5
			
C4—S1—C1—C2	0.0	C11—C12—C13—C14	51.6 (4)
S1—C1—C2—C3	0.0	C13^i^—C12—C13—C14	−45.1 (3)
C1—C2—C3—C4	0.0	C13^i^—C13—C14—C14^i^	180.0
C2—C3—C4—C5	180.0	C12—C13—C14—C14^i^	−123.8 (3)
C2—C3—C4—S1	0.0	C14^i^—C13—C14—C13^i^	180.0
C1—S1—C4—C3	0.0	C12—C13—C14—C13^i^	56.2 (3)
C1—S1—C4—C5	180.0	C13^i^—C13—C14—C15	−127.0 (4)
C3—C4—C5—C6	180.0	C14^i^—C13—C14—C15	53.0 (4)
S1—C4—C5—C6	0.0	C12—C13—C14—C15	−70.7 (4)
C4—C5—C6—C7	180.0	C11—C10—C15—C17	−119.22 (17)
C5—C6—C7—O1	0.0	C9—C10—C15—C17	60.78 (17)
C5—C6—C7—C8	180.0	C11—C10—C15—C17^i^	119.22 (17)
O1—C7—C8—C9	0.0	C9—C10—C15—C17^i^	−60.78 (17)
C6—C7—C8—C9	180.0	C11—C10—C15—C14^i^	16.7 (2)
C7—C8—C9—C10	180.0	C9—C10—C15—C14^i^	−163.3 (2)
C8—C9—C10—C11	180.0	C11—C10—C15—C14	−16.7 (2)
C8—C9—C10—C15	0.0	C9—C10—C15—C14	163.3 (2)
C9—C10—C11—C12	180.0	C14^i^—C14—C15—C17	−150.87 (16)
C15—C10—C11—C12	0.0	C13^i^—C14—C15—C17	131.5 (6)
C9—C10—C11—C16	0.0	C13—C14—C15—C17	166.3 (3)
C15—C10—C11—C16	180.0	C14^i^—C14—C15—C17^i^	−34.77 (18)
C10—C11—C12—C13	−17.4 (2)	C13^i^—C14—C15—C17^i^	−112.4 (5)
C16—C11—C12—C13	162.6 (2)	C13—C14—C15—C17^i^	−77.6 (4)
C10—C11—C12—C13^i^	17.4 (2)	C14^i^—C14—C15—C10	95.42 (9)
C16—C11—C12—C13^i^	−162.6 (2)	C13^i^—C14—C15—C10	17.8 (6)
C11—C12—C13—C13^i^	96.72 (10)	C13—C14—C15—C10	52.6 (4)
C11—C12—C13—C14^i^	17.1 (6)	C13^i^—C14—C15—C14^i^	−77.6 (6)
C13^i^—C12—C13—C14^i^	−79.6 (5)	C13—C14—C15—C14^i^	−42.8 (4)

Symmetry code: (i) *x*, −*y*+1/2, *z*.
